# Timing of starting anticoagulation following decompressive surgery for cerebral vein and sinus thrombosis: An observational study

**DOI:** 10.1177/17474930251341725

**Published:** 2025-04-30

**Authors:** Mariana C Taveira, Sanjith Aaron, Jorge M Ferreira, Jonathan M Coutinho, Patrícia Canhão, Adriana Conforto, Antonio Arauz, Marta Carvalho, Jaime Masjuan, Vijay K Sharma, Jukka Putaala, Maarten Uyttenboogaart, David J Werring, Rodrigo Bazan, Sandeep Mohindra, Jochen Weber, Bert A Coert, Prabhu Kirubakaran, Mayte Sanchez van Kammen, Pankaj Singh, Diana Aguiar de Sousa, José M Ferro

**Affiliations:** 1 Centro de Estudos Egas Moniz, Faculdade de Medicina, Universidade de Lisboa, Portugal; 2 Serviço de Neurologia, Departamento de Neurociências e Saúde Mental, Unidade Local de Saúde Santa Maria, Lisboa, Portugal; 3 Neurology Unit, Department of Neurological Sciences, Christian Medical College and Hospital, Vellore, Tamil Nadu, India; 4 Serviço de Neurologia, Unidade Local de Saúde São José, Lisboa, Portugal; 5 Department of Neurology, Amsterdam University Medical Centers, University of Amsterdam, Amsterdam, The Netherlands; 6 Hospital das Clínicas HCFMUSP, Faculdade de Medicina, Universidade de São Paulo, São Paulo, Brazil; 7 Stroke Clinic, Instituto Nacional de Neurología y Neurocirugía Manuel Velasco Suárez, Mexico City, Mexico; 8 Serviço de Neurologia, Unidade Local de Saúde São João, Porto, Portugal; 9 Departamento de Neurociências Clínicas e Saúde Mental, Faculdade de Medicina da Universidade do Porto, Porto, Portugal; 10 Servicio de Neurología, Hospital Universitario Ramón y Cajal, IRYCIS, Madrid, Spain; 11 Departamento de Medicina, Universidad de Alcalá. Red RICORS, Madrid, Spain; 12 Department of Medicine, Yong Loo Lin School of Medicine, National University of Singapore, Singapore; 13 Department of Neurology, Helsinki University Hospital and University of Helsinki, Helsinki, Finland; 14 Department of Neurology and Medical Imaging Center, University Medical Center Groningen, University of Groningen, Groningen, The Netherlands; 15 Stroke Research Centre, UCL Queen Square Institute of Neurology, London, UK; 16 Faculdade de Medicina, Universidade Estadual Paulista Julio de Mesquita Filho (Unesp), Botucatu, São Paulo, Brazil; 17 Department of Neurosurgery, Post Graduate Institute of Medical Education & Research (PGIMER), Chandigarh, India; 18 Department of Neurosurgery, Steinenberg Clinic, Reutlingen, Germany; 19 Department of Neurosurgery, Amsterdam University Medical Centers, University of Amsterdam, Amsterdam, The Netherlands; 20 Neurosciences Department, Stroke Center, Centro Hospitalar Universitário Lisboa Central -ULS São José, Lisbon, Portugal; 21 Gulbenkian Institute for Molecular Medicine, Lisboa, Portugal

**Keywords:** Cerebral venous thrombosis, dural sinus thrombosis, decompressive surgery, hemicraniectomy, anticoagulation, heparin

## Abstract

**Background:**

Anticoagulation is the mainstay acute therapy for cerebral venous thrombosis (CVT). Decompressive surgery is required in a small minority of patients with large parenchymal lesions and impending herniation, which requires a temporary suspension of anticoagulation.

**Aim:**

The objective of this study was to identify the optimal timing for starting or resuming anticoagulation following decompressive surgery.

**Methods:**

Data were collected from the Decompressive Surgery for CVT Study 2 (DECOMPRESS2), a prospective multinational cohort observational study of 118 patients with severe CVT treated by decompressive surgery. We assessed the frequency of new hemorrhagic and venous thrombotic events from admission to discharge in patients who started or resumed anticoagulation <24 h (early) and ⩾24 (late) following surgery, using propensity score matching and logistic regression. Death and disability were evaluated by the modified Rankin scale (mRS > 2) at discharge and at 1 year follow-up and compared between the two groups.

**Results:**

Of the 90 patients available for analysis, 35 (39%) started or resumed anticoagulation within the first 24 h after surgery while 55 (61%) did so later than 24 h. Overall frequency of patients with new hemorrhagic or venous thrombotic events from admission to discharge was 26.7% (24 patients), without crude or adjusted for the propensity score statistically significant difference between the early and late anticoagulation groups (<24 h, 11 patients, 31%, vs ⩾24 h, 13 patients, 24%; odds ratio (OR): 0.86; 95% confidence interval (CI): 0.24 to 3.04; χ^2^ = 0.33, p = 0.57). The distribution of major hemorrhagic events was also comparable: 8 (23%) bleedings in the <24 h, and 9 (16%) in the ⩾24 h (χ^2^ = 0.24, p = 0.62). No CVT recurred. Two venous thrombotic events occurred in <24 h (6%) and 5 in the ⩾24 h (9%) group. There was no association between anticoagulation timing and death or dependence (mRS 3-6) at discharge (OR: 1.65. 95% CI: 0.30 to 9.01, p = 0.56), or at 1 year follow-up (OR: 2.19, 95% CI: 0.78 to 6.10, p = 0.14).

**Conclusions:**

The results of this cohort study suggest that the timing of anticoagulation therapy following decompressive surgery for CVT does not significantly influence the risk of new bleeding or venous thrombotic events or disability.

## Graphical abstract

**Graphical abstract:**
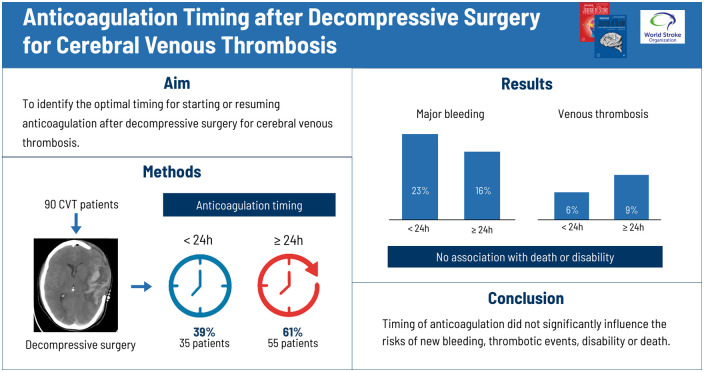

## Background

Cerebral venous thrombosis (CVT) is a special cause of stroke due to thrombosis of the venous dural sinus and/or the encephalic veins, affecting mostly young adults.^1^ The functional outcome after CVT is generally good, but around 5% of CVT patients die in the acute phase, mainly due to fatal transtentorial herniation due to large brain hemorrhagic lesions.^2,3^

Current guidelines advocate anticoagulation as the mainstay therapy in the acute phase, even in the setting of intracranial hemorrhage.^4,5^ In patients with acute CVT and parenchymal lesion(s) with impending herniation, decompressive surgery is recommended as a lifesaving measure.^6^ Decompressive surgery requires a temporary suspension of anticoagulation therapy.

Resuming anticoagulation therapy too early following decompressive surgery may heighten the risk of postoperative bleeding, while postponing anticoagulation can increase the likelihood of recurrent thrombotic events, and of extension of the CVT. Therefore, identifying the optimal time frame for starting or resuming anticoagulation therapy is crucial for the effective and safe management of patients with CVT following decompressive surgery.

Nonetheless, few studies have been conducted to investigate the appropriate timing for anticoagulation initiation or resumption following decompressive surgery.^7^ Most information regarding this matter was acquired secondarily from studies whose main purpose was to describe clinical outcomes following decompressive surgery, leading to imprecise reporting of anticoagulation timings, doses and of new thrombotic or hemorrhagic events.

The primary objective of the present study was to compare the risk of recurrent venous thrombotic events and of bleeding according to the time delay to initiation of anticoagulation after decompressive surgery for CVT.

## Methods

### Study aim

We aimed to report the frequency of new hemorrhagic and of venous thrombotic events from surgery to hospital discharge in a cohort of patients with CVT treated by decompressive surgery, and their association to the timing (initiation or resumption) of anticoagulation following surgery, dichotomized between <24 h and ⩾24 h.

### Design, inclusion and exclusion criteria

The present investigation is a substudy of the Decompressive Surgery for CVT Study 2 (DECOMPRESS2),^6^ a prospective observational multicentre, multinational cohort study of patients with severe CVT, who underwent decompressive surgery. DECOMPRESS2 included consecutive patients with CVT who met the following inclusion criteria: (1) diagnosis of CVT by Magnetic Ressonance (MR) and Magnetic Ressonance Venography (MRV), Computed Tomography Venography (CTV) or Intra-arterial (IA)venography; (2) treated by decompressive craniectomy and/or hematoma evacuation; and (3) written informed consent, according to local laws and regulations. Exclusion criteria comprised patients with (1) CVT diagnosed at exploratory neurosurgery or autopsy, and (2) CVT associated with head trauma or other intracranial disease (e.g. subdural hematoma, ruptured arteriovenous malformation, dural arteriovenous fistula or developmental venous anomaly) with a potential primary indication for decompressive surgery or other type of neurosurgery.

The DECOMPRESS2 cohort included 118 CVT patients from 15 centers in 10 countries in Europe, Asia, and America, who were treated by decompressive surgery between 12/2011 and 12/2019. Patient follow-up lasted for 12 months after surgery. The investigation of risk factors/associated conditions, further investigations, repeated neuroimaging and decisions on treatments, namely initiating or resuming anticoagulation, as well as anticoagulation type and dosage, were left to the discretion of the treating physician. Information on new thrombotic and hemorrhagic events and on start and end of treatments was prospectively recorded.

For the current substudy, we included all patients who had information on the timing of initiation or resumption of anticoagulation after decompressive surgery. Patients with missing or incompletely reported postsurgical anticoagulation timings were excluded (Supplemental Table 3).

### Outcomes

The primary outcome was the combined number of postsurgical (from surgery to hospital discharge) hemorrhagic events (major bleeding and clinically relevant non-major bleeding) as defined by the International Society on Thrombosis and Haemostasis and venous thrombotic events (CVT, deep venous thrombosis (DVT), splanchnic venous thrombosis and pulmonary embolism (PE)).^8^ Recurrent CVT was defined as new neurological symptoms/signs or worsening of previous symptoms/signs and demonstration of new thrombosis of a vein/dural sinus as shown by MR and/or angiography (any modality), in comparison to previous neuroimaging. Secondary outcomes were each of the components of the primary outcome, that is, all postsurgical hemorrhagic events and postsurgical venous thrombotic events. Additional outcomes were new neurosurgery for intracranial hemorrhage, in-hospital death, in-hospital death due to major bleeding, death at 1 year follow-up, functional outcome, as assessed by the modified Rankin scale (mRS) at discharge and at 1 year follow-up, dichotomized by functional independence (mRS 0-2 vs 3-6).

### Statistical analysis

For descriptive analysis, we used frequencies, medians and interquartile range, respectively, for categorical and continuous variables. Baseline variables were compared between the two timing of anticoagulation (<24 h and ⩾24 h), using chi-square statistics, with Yates correction or Fisher’s exact test when needed, and median test, for categorical and continuous variables respectively. Binary outcomes (new hemorrhagic and venous thrombotic events, deaths, dichotomized mRS) were compared between the two groups using chi-square statistics, with Yates correction and Fisher Exact test when indicated. The same statistics were used to explore the association between binary outcomes and the a priori defined confounders and covariates. Because the decision to start heparin <24 h or later is influenced by several presurgery variables, which could be asymmetrically distributed in the two treatment groups, thus introducing selection bias by indication, we used propensity score (PS) methods to minimize such bias. We calculated propensity scores to estimate the probability of each patient to belong to one of the treatment groups, based on a multivariate logistic regression model. The model included the following binary variables: age (above and below the median of the sample); center (for centers with more than 10 included patients); thrombus load (>2 thrombosed sinus), presurgery intracranial hemorrhagic lesion, presurgery heparin, delay between onset and surgery (Table 1, Supplemental material). We then used propensity scores to match patients who received heparin treatment <24 h from surgery with patients who received heparin later than 24 h in a 1:1 ratio within a 0.2 times SD of the logit of PS using nearest neighbor matching according to the caliper method. We planned to compare the outcomes between the two groups in the postmatched sample, using a binary logistic regression model. However, as the number of matches could be too low, we additionally performed a regression analysis with the primary outcome as the dependent variable and timing of anticoagulation after surgery (<24 h vs ⩾24 h) and propensity score as covariates.

Based on the main results of DECOMPRESS2,^6^ for the analysis of death and functional outcome, the following confounders were considered, besides the propensity score: coma and uni- or bilateral fixed dilated pupils. For missing observations of functional outcome at 1 year follow-up, the “last observation carried forward” (discharge or 6 months follow-up) procedure was used. Covariates were checked for collinearity.

Sensitivity analysis was conducted between intravenous (IV) unfractionated heparin (IVUF) and subcutaneous (SC) low-molecular-weight heparin (LMWH) and between therapeutic and prophylactic anticoagulant dosage. These analyses were performed only for the primary outcome.

All statistical analysis was performed using SPSS v28. Statistical significance was set at α < 0.05.

## Results

Of the 118 patients included in DECOMPRESS 2, 11 (9.3%) were excluded due to missing or incomplete information regarding anticoagulation timing. Eight (6.8%) did not resume anticoagulation following surgery and 9 (7.6%) additional patients did not receive any anticoagulation treatment before or after surgery. These 17 patients were not included in the timing of initiation of anticoagulation comparison, but their outcomes are presented in Table 2, Supplemental material. Thus, a total of 90 patients (62 women, median age 38 years) were available for analysis of the timing of initiation of anticoagulation, 54 (60%) of whom started anticoagulation only after surgery, and 36 (40%) who resumed it. There were no statistically significant differences on the main baseline characteristics between those included and excluded from the timing of initiation of anticoagulation analysis (Table 1, Supplemental material), except for bilateral fixed dilated pupils, which were more frequent in the excluded group (23% vs 5%; χ^2^ = 4.61, p = 0.03). The majority of the patients were fully anticoagulated with therapeutic dosages, while 5 patients (5.5%) were prescribed only prophylactic dosages and 17 (18.9%) both prophylactic and therapeutic dosages in succession.

**Figure 1.**
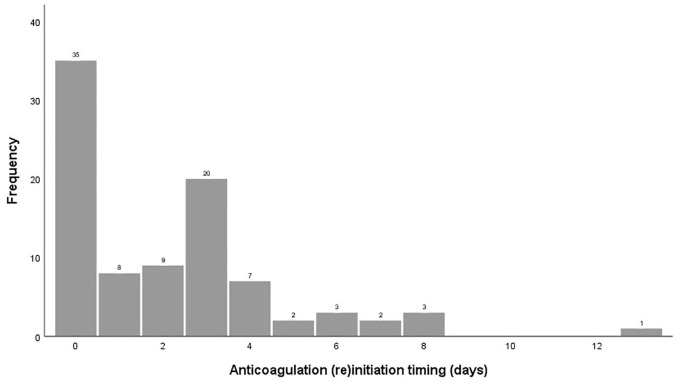
Anticoagulation (re)initiation timing following decompressive surgery.

**Figure 2.**
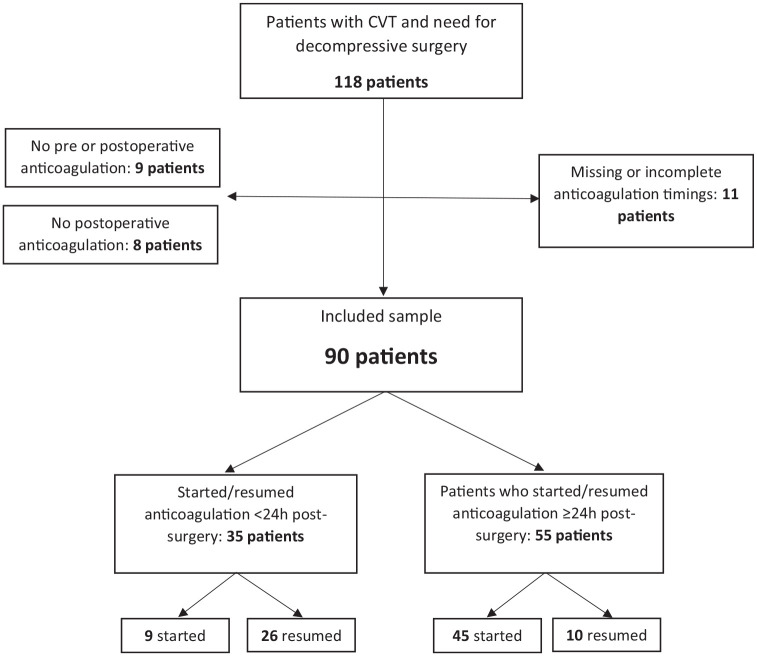
Patient flowchart. CVT: cerebral venous thrombosis.

Thirty-five (39%) patients started/resumed anticoagulation within the first 24 h after surgery, while 55 (61%) initiated/resumed anticoagulation later than 24 h (Figure 1). Figure 2 displays the flow of patients.

Table 1 describes main patient characteristics, comparing the <24 h and the ⩾24 h groups. There was variability among centers on the preferred timing of starting anticoagulation after surgery. Otherwise, the distribution of the other characteristics was overall rather similar between the two groups. There was a statistically significant higher frequency of oral contraceptive users in the <24 h group (44% vs 13%; χ^2^ = 11.1; p = 0.001). Diagnosis-to-surgery interval longer than 1 day (86% vs 48%; χ^2^ = 12.9, p < 0.001) and worsening-to-surgery interval longer than 1 day (50% vs 13%; χ^2^ = 9.8, p = 0.002) were more frequent in the group <24 h. The use of presurgery and postsurgery combined SC and IV heparin was also more frequent in that group (77% vs 18%; χ^2^ = 30.7, p < 0.001; 43% vs 9%; χ^2^ = 14.1, p < 0.001). On the other hand, the use of postsurgery subcutaneous heparin was more frequent in group ⩾24 h (84% vs 51%, χ^2^ = 10.8, p < 0.001).

**Table 1.** Main patient characteristics compared between those who initiated/resumed anticoagulation <24 h and ⩾24 h postsurgery.

Variables	<24 h		⩾24 h	
	n/N	(%)	n/N	(%)
Age > 37 years	22/35	(63)	27/55	(49)
Female	28/35	(80)	34/55	(62)
*Period of inclusion*				
2011-2015	8/35	(23)	17/55	(31)
2016-2020	27/35	(77)	38/55	(69)
*Center (city, country))*				
Vellore, India^a^	8/35	(23)	40/55	(73)
Singapore, Singapore	3/35	(9)	0/55	(0)
São Paulo, Brazil	4/35	(11)	4/55	(7)
Botucatu, Brazil	1/35	(3)	0/55	(0)
Mexico City, Mexico	3/35	(9)	2/55	(4)
Amsterdam, Netherlands^b^	9/35	(26)	3/55	(13)
Groningen, Netherlands	1/35	(3)	0/55	(0)
Porto, Portugal	6/35	(17)	1/55	(2)
Lisboa, Portugal	0/35	(0)	1/55	(2)
Madrid, Spain	1/35	(3)	2/55	(4
Helsinki, Finland	0/35	(0)	1/55	(2)
Reutlingen, Germany	0/35	(0)	1/55	(2)
*Last observation and imaging before surgery*				
Headache	32/35	(91)	50/55	(91)
Aphasia	11/35	(31)	12/55	(22)
Motor deficit	27/35	(77)	36/55	(65)
Seizure	17/35	(49)	27/55	(49)
Coma (GCS < 9)	22/35	(63)	28/55	(51)
Unilateral fixed dilated pupil	9/35	(26)	12/44	(27)
Bilateral fixed dilated pupils	0/35	(0)	4/44	(10)
Number of thrombosed sinus (median)	2		2	
Intracerebral hemorrhagic lesion	31/35	(89)	53/55	(96)
Large intracranial hemorrhagic lesions (⩾6.5 cm)	26/34	(76)	33/55	(60)
*Risk factors/associated conditions*				
Oral contraceptives^c^	15/34	(44)	7/55	(13)
Other prothrombotic drugs	1/34	(3)	7/55	(13)
Pregnancy/puerperium	5/34	(15)	10/55	(18)
Infection	2/34	(6)	4/55	(7)
Malignancy	1/34	(3)	2/54	(4)
Myeloproliferative neoplasm	0/37	(0)	1/54	(2)
Severe anemia	3/34	(9)	7/54	(13)
Thrombophilia, genetic	7/32	(22)	16/49	(33)
Thrombophilia, acquired	2/33	(6)	3/53	(6)
Vasculitis	0/34	(0)	2/54	(4)
Inflammatory bowel disease	1/33	(3)	0/54	(0)
*Treatments*				
Presurgery heparin^d^	27/35	(77)	10/55	(18)
Therapeutic dosage	26/35	(74)	10/55	(18)
Prophylactic dosage	2/35	(6)	0/55	(0)
*Surgery*				
Interval diagnosis-surgery (>1 day)^e^	30/35	(86)	26/54	(48)
Interval worsening-surgery (>1 day)^f^	15/30	(50)	4/31	(13)
Craniectomy only	26/35	(74)	39/55	(71)
Hematoma evacuation only	0/35	(0)	1/55	(2)
Both	9/35	(26)	15/55	(27)
Posterior fossa surgery	0/35	(0)	1/55	(2)
Bilateral hemicraniectomy	3/35	(9)	1/55	(2)
*Anticoagulation after surgery*				
IVUF heparin	2/35	(6)	4/55	(7)
SC LMWH heparin^g^	18/35	(51)	46/55	(84)
Both^h^	15/35	(43)	5/55	(9)
Therapeutic dosage	26/35	(74)	42/55	(76)
Prophylactic dosage	1/35	(3)	4/55	(7)
Both	8/35	(23)	9/55	(16)

n/N—number of patients with condition/number with variable; (%)—percentage within group; IVUF—Intravenous unfractionated heparin; SC LMWH—Subcutaneous low-molecular-weight heparin.

a Chi square = 19.42; p < 0.001.

b Chi square with continuity correction = 5.95; p = 0.014.

c Chi square = 11.13; p = 0.001.

d Chi square = 30.7; p < 0.001.

e Chi square = 12.85; p < 0.001.

f Chi square = 9.78; p = 0.002.

g Chi square = 10.8, p < 0.001.

h Chi square = 14.1, p < 0.001.

### Hemorrhagic and venous thrombotic events after surgery

In total, the combined frequency of patients who suffered new hemorrhagic or venous thrombotic events after surgery in this cohort was 26.7%, 20% hemorrhagic, and 7.8% thrombotic. One patient suffered both hemorrhagic and thrombotic complications (intracranial bleeding and PE) (Table 2).

**Table 2.** Postsurgical bleeding events, new venous thrombotic events, death and functional outcome in patients who started/restarted anticoagulation before or after 24 h after surgery.

Outcomes	⩽24 h		>24 h	
	n/N	(%)	n/N	(%)
All new major bleedings	8/35	(23)	9/55	(16)
New intracerebral hemorrhage	8/35	(23)	9/55	(16)
New major systemic hemorrhage	0/35	(0)	0/55	(0)
Clinically relevant non-major bleeding	2/35	(6)	0/55	(0)
All bleedings	9/35	(26)	9/55	(16)
All new venous thrombotic events	2/35	(6)	5/55	(9)
Cerebral venous thrombosis	0/38	(0)	0/55	(0)
Deep venous thrombosis	0/38	(0)	1/55	(2)
Superficial venous thrombosis	0/38	(0)	1/55	(2)
Pulmonary embolism	2/35	(6)	3/55	(5)
Death				
In-hospital death	7/35	(20)	7/55	(13)
Death at 1 year	1/24	(4)	1/41	(2)
All deaths at last follow-up	11/35	(31)	13/55	(24)
Modified Rankin Scale at discharge				
Independence (0−2)	2/35	(6)	5/55	(9)
Dependency or death (3−6)	33/35	(94)	50/55	(91)
Modified Rankin Scale at 1 year				
Independence (0−2)	10/24	(42)	25/41	(61)
Dependency or death (3−6)	14/24	(58)	16/41	(39)

n/N—number with condition/number with variable.

(%)—percentage within group.

Eighteen patients (20%) suffered new hemorrhagic events: 17 had a new intracranial bleeding (16 intracerebral, 5 subdural, 3 epidural and 2 subarachnoid bleeding), one of whom also experienced a clinically relevant non-major bleeding (leaking surgical wound). One additional patient suffered a non-major clinically relevant bleeding at the tracheostomy site.

The frequency of postsurgical venous thrombotic events was 7.8%, occurring in 7 patients: 5 presented with PE, 1 presented with acute left cephalic vein thrombosis and 1 with left subclavian, brachiocephalic, popliteal, femoral, and iliac vein thrombosis. None had a recurrent CVT. One of the 7 patients with postsurgical venous thrombotic events was a man and one was older than 50 years. In five of these patients, a prothrombotic condition was identified: hematological malignancy, severe anemia, and prothrombotic medication in one; oral contraceptives in two patients, one of whom also had severe anemia; puerperium in the fourth; and pregnancy and genetic thrombophilia in the fifth.

### Comparison of hemorrhagic and venous thrombotic events, in patients who started or resumed anticoagulation <24 h and ⩾24 h after surgery

There was no significant difference between the frequency of new hemorrhagic or venous thrombotic events in the two groups (31% vs 24%; χ^2^ = 0.33, p = 0.57). Propensity score matching generated only 18 out of 35 possible matches, an insufficient number for further statistical comparisons. In regression analysis using propensity score as a covariate there was no statistically significant change in the relative risk of new hemorrhagic or venous thrombotic events associated with starting or resuming anticoagulation <24 h as compared to ⩾24 h after surgery (odds ratio (OR): 0.86; 95% CI: 0.24 to 3.04).

The distribution of major hemorrhagic events was not statistically significantly different between the two groups: 8 (23%) in the <24 h group, 9 (16%) in the ⩾24 h (χ^2^ = 0.24, p = 0.62). All the mentioned patients suffered intracranial bleeding, and one of them also suffered a clinically relevant bleeding from the surgical wound. We found no statistically significant association between the frequency of postsurgical intracranial hemorrhagic events and the presence of presurgical intracranial hemorrhagic lesions (χ^2^ = 0.47, p = 0.49).

Concerning new venous thrombotic events after surgery, two (6%) occurred in group <24 h and five (9%) in group ⩾24 h (χ^2^ = 0.03, p = 0.86).

### Sensitivity analysis

There were no differences in the distribution of the primary outcome, considering only the patients who received IVUF or LMWH or were prescribed therapeutic or prophylactic anticoagulant dosage (Tables 2 and 4, Supplemental material).

### Death and functional independence at discharge and at 1 year follow-up

At discharge, 14 patients were dead, 7 in each group. Only 7 patients were independent (mRS 0-2), 2 (6%) in the <24 h and 5 (9%) in the ⩾24 h. There was no association between anticoagulation timing and death or dependence (mRS 3-6) at discharge (OR: 1.65. 95% CI: 0.30 to 9.01, p = 0.56).

At 1 year follow-up, 2 additional patients were dead, 1 in each group, and 28 more were functionally independent, 8 (33%) in <24 h and 20 (49%) in the ⩾24 h. No statistically significant association was found between anticoagulation timing and death or dependence at 1 year follow-up (OR: 2.19, 95% CI: 0.78 to 6.10, p = 0.14).

## Discussion

Although anticoagulation is the mainstay acute treatment in CVT, current guidelines do not provide recommendations on timing of anticoagulation (re)initiation for patients with CVT who undergo decompressive surgery.^4,8^

Our observational study showed that the combined frequency of hemorrhagic and venous thrombotic events after decompressive surgery was high. Approximately a quarter of CVT patients who underwent decompressive surgery suffered either a hemorrhagic or a venous thrombotic in-hospital event after surgery. The most common complication was intracranial bleeding (18.9%), which was found to be independent from the presence of preoperative hemorrhagic lesions.

On the other hand, we found a relatively lower frequency of postoperative venous thrombotic events (7.8%), particularly CVT recurrence (N = 0). Whether this low proportion is due to anticoagulation is plausible, but cannot be concluded from this study, because we did not have an appropriate control group.

Interestingly, the timing of anticoagulation initiation or resumption did not seem to influence the risk of these complications, as the distribution of both hemorrhagic and thrombotic events was not statistically different between the group of patients who started or resumed anticoagulation before 24 h and those where anticoagulation was postponed to more than 24 h after surgery.

In addition, timing of anticoagulation resumption did not influence in-hospital death or death at 1 year, or functional outcome at discharge and at 1 year follow-up, as evaluated by the mRS.

Previous literature states that therapeutic anticoagulation can be safely initiated or resumed around 48 h postoperatively. This statement is based on a systematic review by Salottolo et al,^7^ which included 15 previous studies and an original case series of four patients, totaling 247 patients. The largest case series in the review, by Zhang et al,^9^ included 58 patients who resumed anticoagulation 48 h after surgery. The same timing was used in a study by Aaron et al.^10^ (44 patients) and Vivakaran et al.^11^ (34 patients). Nonetheless, seven smaller series, with a total of 70 patients, reported resumption of therapeutic anticoagulation as early as 24 h postoperatively,^12–15^ three of which (27 patients) also included the resumption of prophylactic anticoagulation at 12 h postsurgery, followed by therapeutic dose at 24 h.^16–18^ Although most studies described mortality, the frequency of new hemorrhage or progression was not consistently reported in these studies. Most of the studies were intended to describe clinical outcomes following decompressive surgery, and the timing of anticoagulation postsurgery was not their primary aim.

Our results suggest that anticoagulation can probably be safely resumed before 24 h postsurgery, as there were no more bleedings, including intracranial, in this time frame group. The functional outcomes were also similar between the two groups. The observed difference to the previous review can be attributed to the fact that most clinicians are generally hesitant to resume anticoagulation therapy at such an early stage, thereby limiting the number of cases available for analysis and impacting the statistical power within this particular timing subgroup.

Our study has several strengths compared to previous research, as we registered detailed information on all events, outcomes, and complications after surgery and all that information was entered prospectively. Furthermore, our study is based on the largest available cohort of CVT patients who underwent decompressive surgery, which enhances its statistical power.

Our results must be interpreted with caution, because of several limitations of the study, which was an observational study, with no random allocation of time of starting anticoagulation and no blind evaluation of the outcomes. The study has a high risk of bias by indication/contraindication. We used propensity score matching score to minimize the selection bias, related to the variable likelihood of subjects being selected for one of two interval treatment groups. Unfortunately, our sample size was not big enough to generate a large number of matches. We could only adjust for the measured variables, and we could not control for residual bias. Some outcomes, in particular new venous thrombotic events, had a very low frequency, which may not provide sufficient power to identify a statistically significant difference in its frequency between the 2 groups of anticoagulation timing.

Another limitation of this study is that it only considers two time intervals (before and after 24 h postsurgery), whereas in a hospital setting, physicians may choose to delay anticoagulation for longer periods. We chose to use only two time frames, because the sample size of our study did not provide enough numbers to conduct a powered statistical analysis using more time intervals. Although the study was performed in ten countries in three continents, its generalizability is limited in countries which did not participate in the study such as the United States, China, Australasia, and Africa.

In conclusion, our study did not show statistically significant differences in the frequency of venous thrombotic events and of hemorrhagic events between patients who started anticoagulation within or after 24 h from surgery. These results suggest that anticoagulation can probably be safely initiated within 24 h of surgery. However, the high frequency of bleeding events, that can be potentially related to anticoagulation, highlights the need for careful monitoring of patients and individualized decision-making regarding the optimal timing of anticoagulation, in order to decrease complications, and improve patient survival and functional outcomes.

## Supplemental Material

sj-docx-1-wso-10.1177_17474930251341725 – Supplemental material for Timing of starting anticoagulation following decompressive surgery for cerebral vein and sinus thrombosis: An observational study Supplemental material, sj-docx-1-wso-10.1177_17474930251341725 for Timing of starting anticoagulation following decompressive surgery for cerebral vein and sinus thrombosis: An observational study by Mariana C Taveira, Sanjith Aaron, Jorge M Ferreira, Jonathan M Coutinho, Patrícia Canhão, Adriana Conforto, Antonio Arauz, Marta Carvalho, Jaime Masjuan, Vijay K Sharma, Jukka Putaala, Maarten Uyttenboogaart, David J Werring, Rodrigo Bazan, Sandeep Mohindra, Jochen Weber, Bert A Coert, Prabhu Kirubakaran, Mayte Sanchez van Kammen, Pankaj Singh, Diana Aguiar de Sousa and José M Ferro in International Journal of Stroke
