# Supplementary material for: Timing of starting anticoagulation following decompressive surgery for cerebral vein and sinus thrombosis: An observational study
Source: Int J Stroke. 2025 Apr 30;20(10):1225–34. doi: 10.1177/17474930251341725 (PMC12664934; doi:10.1177/17474930251341725)
Supplement: sj-docx-1-wso-10.1177_17474930251341725 – Supplemental material for Timing of starting anticoagulation following decompressive surgery for cerebral vein and sinus thrombosis: An observational study [file sj-docx-1-wso-10.1177_17474930251341725.docx]

**Supplementary material**

**Timing of restarting anticoagulation following decompressive surgery for cerebral vein and sinus thrombosis: an observational study**

Mariana C Taveira, Sanjit Aaron, Jorge M Ferreira, Jonathan M Coutinho, PhD, Patrícia Canhão, Adriana Conforto, Antonio Arauz, Marta Carvalho, Jaime Masjuan, Vijay K Sharma, Jukka Putaala, Maarten Uyttenboogaart, David J Werring, Rodrigo Bazan, Sandeep Mohindra, Jochen Weber, Bert A Coert, Prabhu Kirubakaran, Mayte Sanchez van Kammen, Pankaj Singh, Diana Aguiar de Sousa, José M Ferro, and the DECOMPRESS2 study group

Table 1. Logistic regression to calculate propensity score matching

________________________________________________________________

Variable p value Exp(B)_________

Center, Vellore 0.002 11.376

Center, Amsterdam 0.466 1.924

Age 0.310 0.520

Thrombus load 0.062 0.290

Presurgery intracranial hemorrhage 0.060 10.356

Presurgery_heparin 0.172 0.403

Interval diagnosis-surgery 0.036 0.221

Table 2. Sensitivity analysis: comparison of the distribution of the primary outcome between patients who were treated with intravenous or subcutaneous heparin.

|  | IV heparin | | SC heparin | | Both | | None |
| --- | --- | --- | --- | --- | --- | --- | --- |
| Interval (hours) surgery-heparin | <24 | ≥24 | <24 | ≥24 | <24 | ≥24 | - |
| Number (%) of patients | 2 (6) | 4 (7) | 18 (51) | 46 (84) | 15 (43) | 5 (9) | 17 (14) |
| Number (%) of patients with primary outcome* | 1 (50) | 1 (25) | 5 (28) | 10 (22) | 5 (33) | 2 (40) | 7 (41) |
| Major bleeding |  | | | | | | |
| Intracranial | 1 | 1 | 3 | 7 | 4 | 1 | 5 |
| Systemic | 0 | 0 | 0 | 0 | 0 | 0 | 0 |
| Clinically relevant non-major bleeding | 0 | 0 | 1 | 0 | 1 | 0 | 1 |
| Venous thrombotic events |  | | | | | | |
| Cerebral Venous Thrombosis | 0 | 0 | 0 | 0 | 0 | 0 | 0 |
| Deep venous thrombosis | 0 | 0 | 0 | 1 | 0 | 0 | 0 |
| Superficial venous thrombosis | 0 | 0 | 0 | 1 | 0 | 0 | 0 |
| Pulmonary embolism | 0 | 0 | 2 | 2 | 0 | 1 | 1 |
| Splanchnic venous thrombosis | 0 | 0 | 0 | 0 | 0 | 0 | 1 |

*Fisher Exact Test <24h vs. ≥24h: IV heparin, p=1.00; SC heparin, p=0.83

Table 3. Main characteristics of patients included in and excluded from the timing of initiation of anticoagulation analysis

Variables Included Excluded

n/N (%) n/N (%)

____________________________________________________________________________

Age >37 years 49/90 (54) 11/28 (39)

Female 62/90 (69) 18/28 (64)

Last observation and imaging before surgery

Coma (GCS <9) 50/90 (56) 20/28 (71)

Unilateral fixed dilated pupil 21/79 (26) 4/22 (18)

Bilateral fixed dilated pupils 4/79 (5) 5/22 (23)

Intracerebral hemorrhagic lesion 84/90 (93) 23/26 (89)

Large intracranial hemorrhagic lesions (≥6,5 cm) 59/89 (66) 19/25 (76)

Interval diagnosis-surgery (>1 day) 56/89 (63) 16/27 (59)

Interval worsening-surgery (>1 day) 19/61 (31) 3/21 (14)

Pre-surgery heparin 37/90 (41) 14/24 (58)

Therapeutic dosage 36/90 (40) 12/24 (50)

Prophylactic dosage 2/90 (2) 3/24 (13)

Surgery

Craniectomy only 65/90 (72) 16/28 (57)

Hematoma evacuation only 1/90 (1) 2/28 (7)

Both 24/90 (27) 10/28 (36)

Posterior fossa 1/90 (1) 1/26 (4)

Bilateral hemicraniectomy 4/90 (4) 4/26 (15)

n/N – number of patients with condition/number with variable

(%) – percentage within group

All 2x2 frequency comparisons were statistically non-significant, except for bilateral fixed dilated pupils (*p*=0.03)

Table 4. Sensitivity analysis: comparison of the distribution of the primary outcome between patients who receive therapeutic or prophylactic heparin dosages.

|  | Therapeutic | | Prophylactic | | Both | |
| --- | --- | --- | --- | --- | --- | --- |
| Interval (hours) surgery-heparin | <24 | ≥24 | <24 | ≥24 | <24 | ≥24 |
| Number (%) of patients | 26 (74) | 42 (76) | 1 (3) | 4 (7) | 8 (23) | 9 (16) |
| Number (%) of patients with primary outcome* | 8 (31) | 7 (17) | 1 (100) | 2 (50) | 2 (25) | 4 (44) |
| *Major bleeding* |  |  |  |  |  |  |
| Intracranial | 5 | 6 | 1 | 0 | 2 | 3 |
| Systemic | 0 | 0 | 0 | 0 | 0 | 0 |
| Clinically relevant non-major bleeding | 1 | 0 | 1 | 0 | 0 | 0 |
| *Venous thrombotic events* |  |  |  |  |  |  |
| Cerebral Venous Thrombosis | 0 | 0 | 0 | 0 | 0 | 0 |
| Deep venous thrombosis | 0 | 0 | 0 | 1 | 0 | 0 |
| Superficial venous thrombosis | 0 | 1 | 0 | 0 | 0 | 0 |
| Pulmonary embolism | 2 | 1 | 0 | 1 | 0 | 1 |
| Splanchnic venous thrombosis | 0 | 0 | 0 | 0 | 0 | 0 |

*Fisher Exact Test <24h vs. ≥24h: therapeutic, p=0.14; prophylactic, p=1.00
